# Reflex “toothbrushing” epilepsy: Seizure freedom after focal ablation assisted by ictal fMRI

**DOI:** 10.1002/epd2.70027

**Published:** 2025-04-08

**Authors:** Michael Ginevra, John Archer, Kristian Bulluss, Chris Tailby, Graeme D. Jackson, David N. Vaughan

**Affiliations:** ^1^ Department of Neurology Austin Health Melbourne Victoria Australia; ^2^ Department of Medicine University of Melbourne Melbourne Victoria Australia; ^3^ Department of Neurosurgery Austin Health Melbourne Victoria Australia; ^4^ Department of Surgery University of Melbourne Melbourne Victoria Australia; ^5^ Florey Institute of Neuroscience and Mental Health Melbourne Victoria Australia; ^6^ Department of Neuropsychology Austin Health Melbourne Victoria Australia

**Keywords:** fMRI guided stereoelectroencephalography, reflex seizures, toothbrushing induced seizures

## Abstract

A 22‐year‐old female presented with drug‐resistant focal motor seizures with onset at age 14. This manifested as daily episodes of right facial dystonia triggered by toothbrushing, but also by eating, talking, and strenuous exercise. On ictal scalp EEG, there was low‐voltage fast activity over the left pericentral area. Structural MRI did not identify a definite lesion. Functional MRI (fMRI) of a reflex seizure, as well as task‐based fMRI during toothbrushing, both demonstrated focal activation at the left low pericentral cortex. Stereoelectroencephalography (sEEG) showed recurrent ictal trains of focal spiking concordant with the fMRI activation. Radiofrequency (RF) thermocoagulation was applied at the posterior bank of the left low pre‐central gyrus, with post‐operative MRI confirming small ablative lesions immediately deep to the ictal fMRI activation, and the patient remains seizure‐free more than 3 years after this treatment. Toothbrushing epilepsy is a rare form of reflex epilepsy where seizures are induced by toothbrushing. In this unique case, ictal fMRI assisted targeting of the sEEG implantation, to confirm seizure onset and enable minimally invasive treatment via RF thermocoagulation, resulting in seizure freedom.

**Key points**

- Toothbrushing epilepsy is a rare form of reflex epilepsy where seizures are induced by toothbrushing.
- Ictal fMRI can assist in targeting sEEG implantation to confirm seizure onset and enable minimally invasive treatment.
- In this case, ictal fMRI of a typical reflex seizure was used to guide sEEG, which subsequently enabled successful treatment using highly focal radiofrequency thermocoagulation.
- The use of RF‐TC to achieve seizure freedom underscores the effectiveness of this combined approach for precisely targeted treatment.

## 1 INTRODUCTION

Toothbrushing epilepsy is a rare “reflex” epilepsy,^1^ where the nature of the invoking stimulus implies seizure onset in or near the mouth area of the sensorimotor strip. Seizures in toothbrushing epilepsy have been reported to be caused by lesions in either the pre‐ or post‐central gyrus.^2^, ^3^, ^4^

Functional MRI (fMRI) can be used in the pre‐surgical assessment of patients with focal epilepsy to help guide surgical exploration.^5^ fMRI uses blood‐oxygen‐level‐dependent (BOLD) signal as an indirect measure of neuronal activity. Combining this with temporal information from simultaneous EEG permits spatial localization of fMRI changes time‐locked to epileptic events.^6^ fMRI during seizures (ictal fMRI), either with or without EEG, is captured less commonly because events are infrequent, and motion artifacts can degrade image quality. Reflex seizures are a situation in which, given the ability to control timing of seizures, ictal fMRI is feasible.^7^, ^8^

Stereoelectroencephalography (sEEG) is often used in the pre‐surgical evaluation of focal epilepsy and can be coupled with radiofrequency thermocoagulation (RF‐TC). This can render select patients seizure‐free, especially in the presence of a focal structural lesion.^9^ In RF‐TC, very high‐frequency (radio band) current is passed between two electrode contacts, leading to the heating of local tissue. Only a small volume of tissue can be lesioned by this technique, typically 5‐mm diameter (100 mm^3^)^10^, ^11^ placing critical importance on precisely defining the seizure onset zone.^12^ In cases with a defined focal lesion such as subcortical nodular heterotopia, long‐term seizure freedom rates can be as high as 70%, whereas in cases without a clear structural lesion, long‐term seizure freedom rates are below 10%.^12^, ^13^

We present a case of toothbrushing epilepsy where ictal fMRI of a typical reflex seizure was used to guide sEEG, which subsequently enabled successful treatment using highly focal radiofrequency thermocoagulation.

## 2 CLINICAL SUMMARY

A 22‐year‐old left‐handed female presented with toothbrushing‐induced focal aware seizures since age 14 years. Seizures were reliably induced by brushing the right molars and began with right facial dystonia. Longer attacks also involved right shoulder dystonic abduction. Awareness was preserved throughout. Seizures could also occur with eating and talking. From age 15, she began having seizures spontaneously from sleep. Family history for epilepsy was negative. Motor and developmental milestones were normal. At the time of surgical evaluation, the patient was having daily events with up to 8 seizures per day, approximately half from brushing her teeth but also spontaneously or with other oral or jaw stimulation, including eating. Seizures were resistant to medications, continuing despite Lacosamide 100 mg twice daily, Topiramate 100 mg twice daily, and Levetiracetam 500 mg twice daily. She had previously trialed Carbamazepine and Clonazepam without benefit.

One week of video scalp EEG monitoring captured 25 typical focal aware seizures, including six seizures induced by toothbrushing. These were triggered with the use of a manual or electric toothbrush held in either hand, implying mouth‐sensory stimulation was the primary trigger. Ictal EEG began with low‐voltage fast activity over the left pericentral region (C3), before spreading frontally (F3/F7). No interictal discharges were seen. Structural 3 T MRI of the brain was reviewed by a sub‐specialist Neuroradiologist in the epilepsy pre‐surgical multidisciplinary meetings, and no definite epileptogenic lesion was seen.

Neuropsychology testing revealed impaired verbal fluency on a background of normal verbal memory and intellect, suggestive of left dorsolateral prefrontal dysfunction. Bedside testing suggested subtle impairment in fine motor function of the right hand.

## 3 IMAGING FINDINGS

### 3.1 fMRI acquisition

Siemens 3 T Prisma‐fit scanner with a 32‐channel head coil was used. BOLD‐weighted images were acquired using the CMRR multiband multiecho echo‐planar imaging sequence^14^ (TR 900 ms, 3 echoes, 3 mm^3^ isotropic voxels, 44 axial slices, multiband 4, GRAPPA 2) with concurrent respiratory‐belt recording. fMRI data were pre‐processed using fMRIprep 1.5.8,^15^ including susceptibility distortion correction^16^, ^17^, ^18^ and registration to the patient's T1‐weighted image.^19^ GLM analysis was conducted in SPM12, using task and seizure blocks convolved with the canonical hemodynamic response function, temporal and dispersion derivatives. Nuisance regressors included mean white‐matter and CSF signals, 6 motion regressors, respiratory‐belt regressors,^19^ and censoring of high‐motion time points (FD >.9 mm). A voxel‐wise threshold *p* < .001 with family‐wise cluster correction *p* < .05 was applied.

### 3.2 Ictal fMRI (Figure 1A)

**FIGURE 1:**
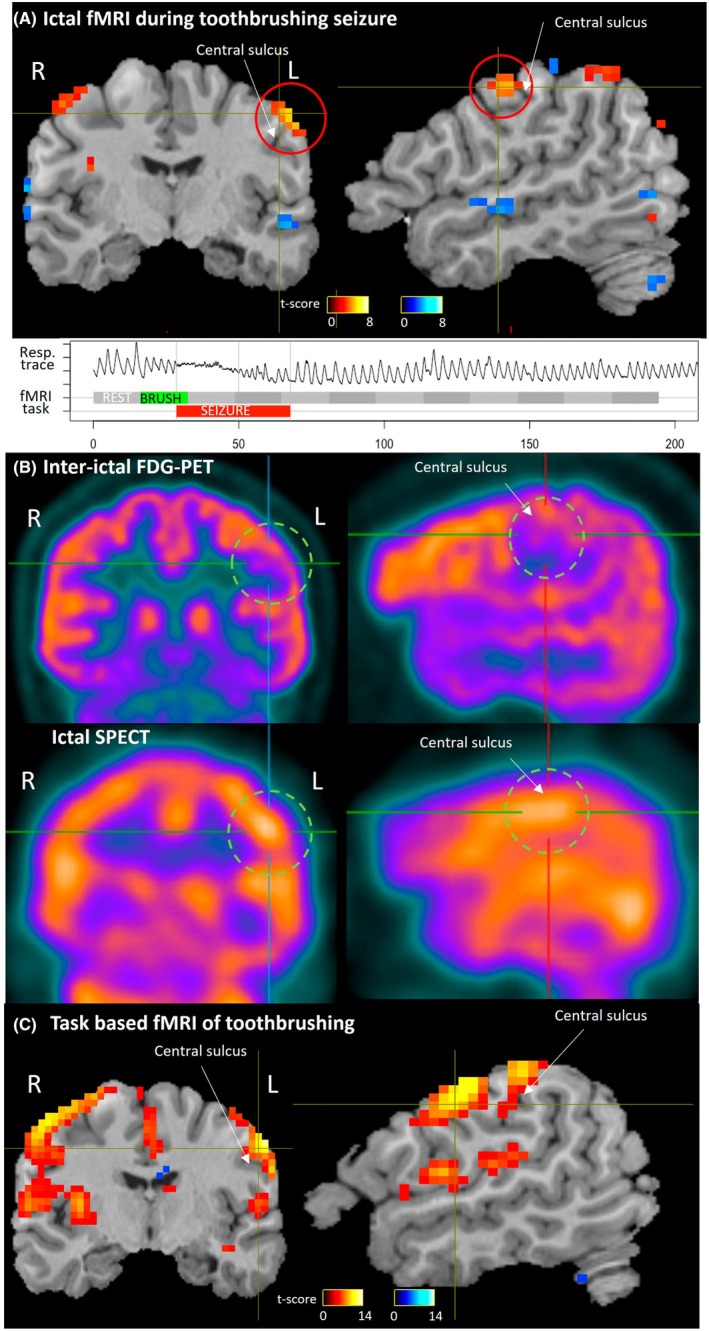
(A) fMRI activation during typical reflex seizure induced during task‐based fMRI. Seizure timing was detected by the change in respiration and direct observation, relative to the planned experimental task timing. (B) FDG‐PET showing regional focal hypometabolism (green dashed circle); ictal SPECT showing area of increased perfusion (green dashed circle). (C) Cortical activation during toothbrushing‐task fMRI (of right molars) over 5 runs where no seizure was triggered.

A typical seizure was triggered during the first run of toothbrushing. Twelve seconds after the start of brushing, there was a respiratory pause (Figure 1A). An observer noted behavioral arrest, failure to resume brushing on cue, and later subtle facial clonus. Total seizure duration was 39 s. The patient recovered over a few minutes and was able to continue with the in‐scanner toothbrushing task. Seizure‐related fMRI activation was maximal at the left low precentral gyrus (peak *t*‐score 5.9; Figure 1A).

### 3.3 PET and SPECT (Figure 1B)

FDG‐PET showed hypometabolism in the left low pericentral region (Figure 1B). Ictal SPECT with early tracer injection (3 s into a typical 32‐s induced seizure) showed a minor perfusion increase also in the left pericentral region (Figure 1B). Both these studies are broadly supportive of the ictal fMRI localization, albeit both with a more diffuse and regional pattern.

### 3.4 Brushing‐task fMRI (Figure 1C)

Six runs of the toothbrushing task were performed, with blocks of brushing (16.2 s) alternating with rest (16.2 s), cued by a visual stimulus. Brushing was performed on the right molars using the left hand, to produce hand‐motor and mouth‐sensory activation at opposite hemispheres. Task activation was seen bilaterally over the primary sensorimotor areas and midline supplementary motor area, including at the right‐hemisphere hand‐motor area reflecting the brushing movement (peak *t*‐score > 14; Figure 1C). However, the key result was activation over the left precentral gyrus and in the superior bank of the left Sylvian fissure, corresponding to the left sensorimotor cortex mouth area. This is the same location activated during ictal fMRI, indicating that mouth‐sensory stimulation from toothbrushing is concordant with, and can be inferred to drive, the seizure focus.

### 3.5 Structural MRI (Figure 2A)

**FIGURE 2:**
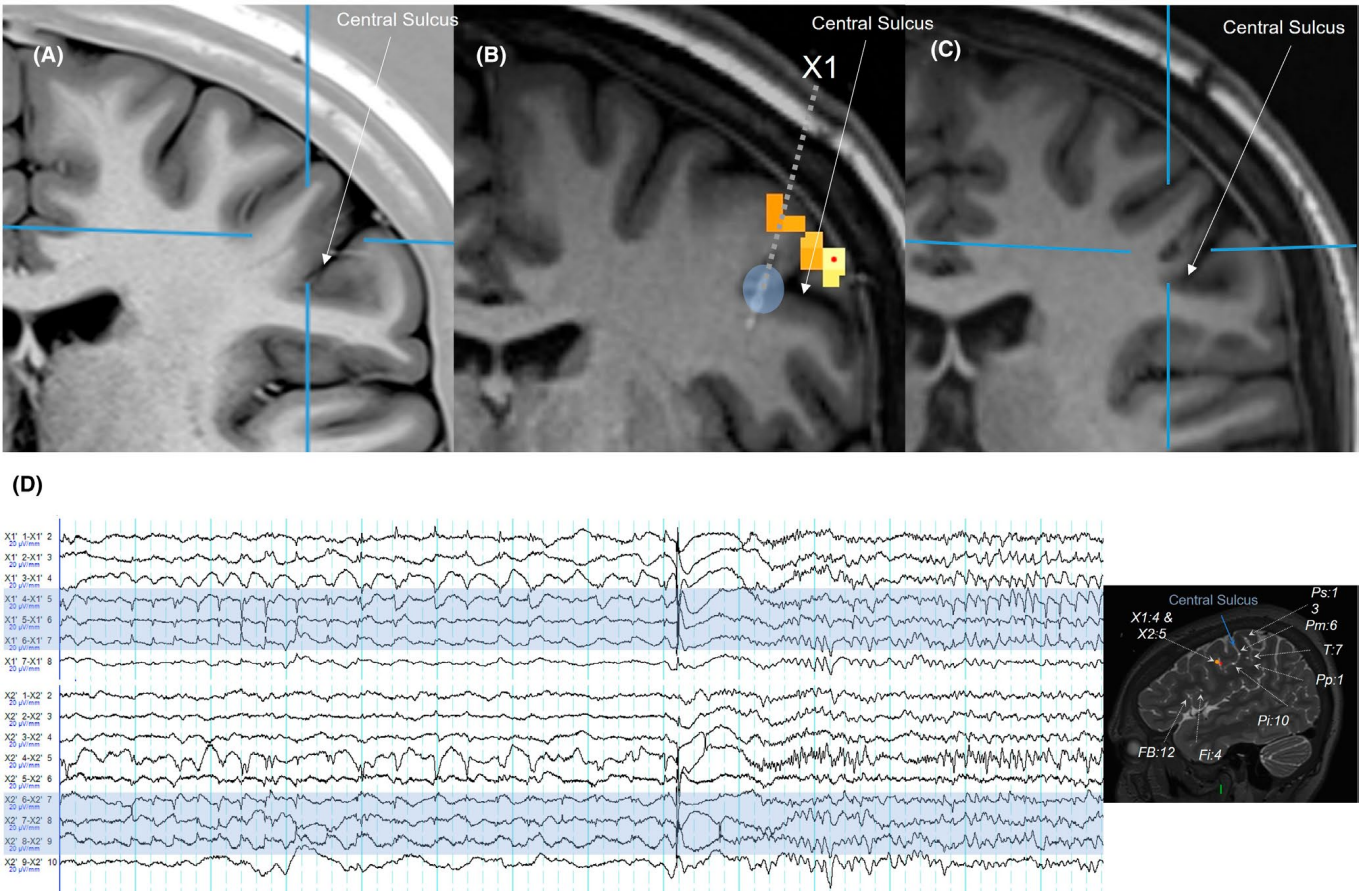
(A) Pre‐operative imaging. T1w inversion recovery (blue lines indicate suspicious band arising from sulcal depth); (B) location of crossing electrodes (X1, X2) (blue shading) in comparison with fMRI activity (yellow highlight); (C) T1‐weighted MPRAGE image 3 months following RF‐TC shows the area of ablation (blue lines). (D) sEEG recording of two crossing electrodes (X1/X2) showing a typical electrographic seizure with pre‐ictal spiking followed by slow wave transient with emerging fast activity. The key crossing contacts are highlighted with blue and correspond to the blue shaded region in panel (B). Insert shows location of key electrode contacts around the region of interest.

Close inspection of the precentral gyrus adjacent and immediately deep to the ictal fMRI activation identified a subtle linear band arising from the depths of the anterior bank of the central sulcus, visible on T1‐weighted and FLAIR imaging. This was reported by an experienced neuroradiologist as very suspicious but not diagnostic for a bottom‐of‐sulcus dysplasia (BOSD) and at the limits of MRI resolution (Figure 2A). Further acquisition of high‐resolution (2 NEX) 3‐D FLAIR sequences as well as 3D pre‐ and post‐contrast T1‐weighted imaging also demonstrated the subtle linear band but again was not considered diagnostic.

## 4 STEREOELECTROENCEPHALOGRAPHY

Nine sEEG electrodes with a total of 100 contacts (DIXI Medical, Marchaux‐Chaudefontaine) were implanted in the left hemisphere, including the low left pericentral region, pre‐ and post‐central gyrus, frontal operculum, and inferior frontal regions. Electrodes were targeted at the presumed tooth/mouth‐sensory areas, as well as cortical regions identified by ictal fMRI, the sensory activation on brushing‐task fMRI, and PET/SPECT findings. A pair of electrodes (X1 and X2) was targeted to intersect at the sulcal fold on the posterior bank of the low precentral gyrus, the region suspected to be at the BOSD, identified from the ictal fMRI study and suspicious structural imaging. Sampling frequency was 1000 Hz.

Multiple electrographic seizures were recorded, although no spontaneous clinical seizures occurred. The recorded seizures had a characteristic pattern of pre‐ictal spiking followed by a slow DC shift with emerging low‐voltage fast (Figure 2D) with greatest amplitude at the middle contacts of the intersecting electrodes X1/X2. Interictal discharges were captured in a similar distribution and typically showed a latency of 10 msec from X1/X2 to other electrodes along the central sulcus. High‐frequency oscillations (HFOs, i.e., >80 Hz) were not detected. These runs of interictal discharges and the patterns of the electrographic seizures are both characteristic of a focal cortical dysplasia (FCD).^20^

Stimulation studies on day 5 produced a typical seizure from the depths of the suspicious sulcus (50 Hz; 5 s; 1 mA; 0.5 ms pulse width). Stimulation also confirmed the expected functional anatomy, with lip/jaw motor and sensory function congruent with activation on the brushing‐task fMRI study. Face/throat, then arm, sensorimotor function was localized more superiorly.

## 5 RADIOFREQUENCY THERMOCOAGULATION AND CLINICAL OUTCOME

Thermocoagulation was performed in two stages, 2 days apart, under continuous sEEG recording. The initial RF‐TC (Cosman G4 via DIXI junction box, 50 V, 30–60 s) was applied to contacts showing maximal interictal spiking at the depths of the sulcal fold at the posterior bank of the precentral gyrus (X1:3–4 and X2:5–6). This blocked the most active spiking, but ongoing activity was noted more toward the cortical surface and just posterior to this region. Therefore, a second‐stage thermocoagulation was performed to target contacts X1:5–7 and X2:6–8. Electrodes were removed immediately after the second RF‐TC. Transient mild right facial weakness was noted, which fully resolved after 4 weeks.

MRI brain performed 3 months post‐sEEG showed small areas of T2‐weighted/FLAIR hyperintensity consistent with the RF‐TC, with the more anterior of these congruent with the region of the presumed FCD (Figure 2C). Topiramate monotherapy was continued for 6 months before being weaned over 3 months, ceasing at 9 months following RF‐TC due to patient preference. At follow‐up 3.5 years following the RF‐TC, the patient had no further reflex seizures and no spontaneous seizures. She had completed university studies, was working, driving a car, and able to brush her teeth.

## 6 DISCUSSION

In this patient with toothbrushing epilepsy, ictal fMRI during a reflex seizure gave spatial localization which subsequent implantation confirmed as the epileptic focus. A highly focal ablation led to seizure freedom.

Reflex epilepsies describe an epilepsy syndrome where all seizures are triggered by a specific stimulus. If other seizures occur, then the epilepsy syndrome is defined by the other seizure types present, with the triggered seizures referred to as reflex seizures.^21^, ^22^ So in our case, this was focal epilepsy with reflex seizures. Reflex seizures are present in 4%–7% of patients living with epilepsy.^22^, ^23^ Different reflex epilepsies include idiopathic photosensitive occipital lobe epilepsy, hot water epilepsy, reading epilepsy, and musicogenic epilepsy. These “pure” reflex epilepsies can run in families, indicating a genetic underpinning, although common specific genes have not been identified.^23^

The area of maximal cortical BOLD activity during a seizure highlighted a specific gyrus as an area of interest and was supported by task‐based fMRI to localize gum sensory areas, PET, SPECT, clinical correlation, and a suspicious appearance on structural MRI. The ictal fMRI localization became the primary target for sEEG implantation, along with other regions expected to be involved in mouth motor and sensory function. We considered both the sEEG electrographic signature of recurrent spiking and the subtle structural MRI features to be consistent with a type of FCD known as a BOSD, although a limitation of RF‐TC is the absence of tissue to confirm this implied pathology. The subtle linear band was not seen during the initial review of imaging, highlighting the importance of repeated multidisciplinary review of imaging.

The strong association between sensory stimulus to the lower right side of the mouth and focal seizures suggested involvement of the mouth and face sensory cortex in seizure generation. This led to the initial hypothesis that the causative lesion would lie in the post‐central gyrus. Indeed, gum sensory areas in the post‐central gyrus showed fMRI activation during brushing, but electrodes in this location did not demonstrate epileptic activity. In fact, ictal fMRI and sEEG demonstrated the epileptic focus in the precentral gyrus (primary motor cortex). We interpret these activation patterns as reflecting an epileptogenic functional network between this patient's precentral gyrus/BOSD and mouth‐sensory cortex. We note there is strong interconnectivity between the precentral gyrus and post‐central gyrus,^24^ and prior reports of toothbrushing epilepsy have found causative lesions in either pre‐ or post‐central gyrus.^1^, ^2^, ^3^, ^4^

There are several ways in which fMRI is a highly complementary technology to the practice of sEEG. When a known action can activate the epileptic network, or a reflex seizure can be reliably triggered inside the scanner (as in this unique case), fMRI can generate a plausible hypothesis for the seizure onset zone at moderate‐to‐high spatial resolution. In the more common scenario when scalp interictal discharges are detectable, which was not possible for this case, simultaneous EEG‐fMRI can be a powerful tool to generate a hypothesis for the interictal spike onset zone.^9^ Such fMRI‐generated hypotheses are best tested using sEEG, as it can target the proposed foci precisely, and from a physiological perspective samples neural activity more directly. Such approaches are particularly beneficial in cases that are equivocal or absent for structural epileptogenic lesions.

In summary, this case demonstrates the value of fMRI in refining the hypothesis for sEEG placement in reflex epilepsy and in identifying the local seizure network. The use of RF‐TC to achieve seizure freedom underscores the effectiveness of this combined approach for precisely targeted treatment.

## AUTHOR CONTRIBUTIONS

MG, JA, and DNV conceptualized and designed the report, wrote the manuscript, and were responsible for all stages of the report. GDJ was the patient's main physician, provided financial support from his research funds, and provided a valuable review of the manuscript. KB was the neurosurgeon who performed the implantation. JA was the attending doctor for the patient and was the planning and reporting physician for the stereoelectroencephalography. DNV, GDJ, and CT designed, analyzed, and reported the fMRI. All authors read and approved the final manuscript.

## CONFLICT OF INTEREST STATEMENT

None of the authors have any conflict of interest to disclose. We confirm that we have read the Journal's position on issues involved in ethical publication and affirm that this report is consistent with those guidelines.

**Test yourself**

1. What role can radiofrequency thermocoagulation (RF‐TC) play in stereoelectroencephalography (SEEG)?
2. What are some of the difficulties with ictal fMRI?
3. In which of the following reflex epilepsies is MRI most likely to show a significant structural brain abnormality, rather than being attributable to a familial or genetic cause:
  - Bathing epilepsy
  - Startle induced epilepsy
  - Photosensitive epilepsy
  - Reading epilepsy
*Answers may be found in the* S1.

## Supporting information


Appendix S1

## Data Availability

The data that support the findings of this study are available on request from the corresponding author. The data are not publicly available due to privacy or ethical restrictions.
