# Supplementary material for: Reflex “toothbrushing” epilepsy: Seizure freedom after focal ablation assisted by ictal fMRI
Source: Epileptic Disord. 2025 Apr 8;27(3):457–63. doi: 10.1002/epd2.70027 (PMC12203305; doi:10.1002/epd2.70027)
Supplement: Supplementary file 1 — Appendix S1 [file EPD2-27-457-s001.docx]

Answers

1. Answer: RF-TC provides a minimally invasive therapy option utilizing the spatial information directly measured by SEEG. In some cases with very focal lesions, it can be effective in rendering patients seizure free. It also can provide prognostic information as a transient period of seizure freedom is positively associated with seizure freedom followed resection in the same area.

2. Answer: ictal fMRI is limited by multiple factors including motion artifact, safety concerns and infrequency and unpredictability of seizures. Reflex seizures provide a unique opportunity to perform ictal fMRI studies in a controlled way.

3. Answer:   Startle induced seizures most often occur in children with structural brain abnormalities, which may be congenital or acquired, for example, in the perinatal period.
